# Investigation of sustainability embodied in existing buildings: a case study of refurbishment adopted in a Chinese contemporary building

**DOI:** 10.1038/s41598-021-96687-9

**Published:** 2021-08-26

**Authors:** Yi Huang, Chong Xu, Yufan Xiao, Bart Dewancker

**Affiliations:** 1 Department of Mechanical and Electrical Engineering, Qingdao Agricultural University, Qingdao, China; 2 Graduate School of Environmental Engineering, University of Kitakyushu, Kitakyushu, Japan; 3 College of Architecture and Urban Planning, Qingdao University of Technology, Qingdao, China; 4 Architectural Association School of Architecture, London, UK

**Keywords:** Civil engineering, Environmental sciences, Engineering

## Abstract

The Liyuan courtyard buildings are considered as contemporary architectural symbols of the spirit in Qingdao, China. The sustainability potentials embodied in the building is evaluated by building performance simulations analysis based on field investigation in this case study. Two models with optimization refurbishment were made through building simulation software. One model with façade supplemented in the insulation layers of the envelope walls and the other model with further upgrade with consideration of recycling materials mixed were discussed and estimated with building performance simulation method. The energy performance in the building and both scenarios designed can improve the energy efficiency, while the advanced model could achieve better result in the building energy behavior dramatically. Technologies innovation are proved to be good tools to improve energy performance the existing buildings by renovation actions such as insulation improvement and so on. It is concluded the sustainability regain its authentic appearance while achieve energy efficiency embodied within contemporary buildings through adaptational renovation strategies. Multicriteria considerations might influence the balanced between different factors when making decisions in the building restoration project, it is also expected to empower the fresh glory in the development of building protection and restoration.

## Introduction

Climate changes and environmental issues have drawn concerns by many countries in consensuses with developing sustainable measures to overcome the crisis in energy shortage and living ecology. It comes up with an agreement on meeting human development goals while simultaneously sustaining the ability of natural systems to provide the natural resources and ecosystem services on which the economy and society depends^1^. The United Nations set 17 sustainable development goals as the blueprint for all the member nations to achieve a better sustainable future. They list the global challenges to overcome, including those related to poverty, inequality, climate change, peace and justice, especially environmental degradation. The 17 Goals are planned to be achieved by 2030 with no one behind^2^ (2021.7.31). As the largest energy consumption resource, the global architecture, engineering and construction (AEC) industry is also responsible for providing the social and economic tenement to the global population, including preserving or retrofitting the buildings and infrastructure already in use. In 2010, Meggers et al.^3^ appraised the true capability of CO_2_ emissions reduction from buildings sector covering challenges of cutting emissions from building construction, operation and maintenance, throughout the whole life cycle. Mazria concluded in 2007^4^ that once taking the building’s whole life cycle into consideration, more than half of the emissions are relevant to building sector. Roodman and Lenssen evaluated that 35% of energy in the world are used by buildings, while they are directly responsible one third of global emissions with the certain consumption level^5^.

Various strategies have been developed to achieve better living environment with sustainable goals. The U.S. Green Building Council committed to a sustainable, prosperous future with LEED rating system, which is recognized as the leading system as sustainable solution to the buildings sector development. The system enables the buildings and communities to regain and sustain the health and vitality of all life within a generation, by the way buildings and communities are designed, built and operated, to achieve an environmentally and socially responsible, healthy, and prosperous environment that improves the quality of life^6^. In Europe, various member nations established their own low-energy building standards under the European Union's management guidance, in order to upgrade the building standard towards zero-energy goals^7^. In Japan, there are policies in control and regulatory; economic-based, fiscal and information actions to realize building energy saving, with purpose to realize zero energy goals as the average for new housing by 2030^8^. Compared to Japan, China is suffering more obstacles such as inefficient enforcement, insufficient levels of information and awareness and immature financial regulation system etc.^9^. In China, under the premise of sustained and stable economic growth in recent years, the proportion of building energy consumption is still likely to continue to rise. To harness the energy potential of existing buildings is a mile stone to meet the targets for a low carbon economy in 2050^10^.

The new trend of scale expansion and population concentration in urban development threaten the global energy balance and the recovery from environmental pollution. Insufficient natural resource and global warming have become new international concerns, which encouraged new innovation as positive solution to keep the global balance between natural and human society. The architecture, engineering and construction (AEC) industry sector is believed to be responsible for considerable amount of global energy demand resulting in a significant negative environmental impacts^3^, they are evaluated to be responsible for 35% of direct emissions with the certain consumption level^5^.

These contemporary buildings exist not only as the representative symbol of the history, but also a positive cultural resource for the future generations. Reasonable refurbishment gained more attention and accepted as an urgent issue as the practical solution in sustainable development in architectural industry^11^. Protection and restoration might bring new concepts in conservation which is essential as contributor to the reduction in environmental impacts. It is of great significance that appropriate approaches are carried out to preserve the whole body in consistent with reduction of general energy consumption applying impacts on the natural environment.

### The renovation of the existing buildings and indicators for sustainability

The energy savings potential by retrofitting existing buildings is a milestone to meet the targets of a Low Carbon Economy. The construction industry has been evolving to embrace sustainability. This has highlighted the necessity to inspect sustainable performances throughout the post-construction building lifecycle. How to implement the retrofit of an existing building efficiently, furthermore, to evaluate the energy performance for low energy goals in environmental impacts is really the problem in the building sector. Considering specific active optional alternatives, it might be an active solution to those 100 years old buildings to overcome the energy hog problem. Deep assessments towards architectural heritages upgrades can be the great opportunities in which the users and residents could face the problem directly. Reasonable retrofit of existing buildings and keep the budget balance would be another option. As such, it relies more on the process, which can be adapted and optimized, than on the results.

A sustainability analysis of building renovation can include many factors; the energy performance, material efficiency, environmental impact, durability, affordability, and social benefit^12^. While the sustainability assessment of buildings and renovation should be based on a lifecycle analysis. Technical performance indicators are added to the environmental performance indicators in a sustainability assessment. Durability of renovation measures is one example of a technical performance indicator. Durability of a building envelope component depends on more factors such as constructional and material properties, maintenance and climate robustness. In a sustainability perspective, the economic performance should be evaluated as life cycle cost^13^. There are many methods are described in the literature as decision making support tools for sustainability assessment^14,15^. Quantitative multicriteria models are the engineering approach for sustainability evaluation. Models with linear functions in the simulation tools on thermal comfort and environmental impact potential are used to evaluate renovation measures in the energy production and environmental^16–19^.

Birgit^13^ made sustainability assessment of zero energy renovation of a Norwegian dwelling based on the standard from the British Institute for sustainability, which published an iterative method as a classical retrofit guide. Brito^20^ took his perspectives on promotion the renovation alternative from a single heritage house into the whole neighborhood scale, which is quite an collective action by accumulating every single effort to achieve the common aim. The author agrees with the iterative method of the sustainability assessment on energy saving, because the whole process itself is complicated involved many factors to think about, meanwhile, different stakeholders would concentrate on their concerned points subjectively. The author also argues that different perspective can provide more possibility on the sustainability analysis of energy saving potential. The uncertainty of the human factor might result in variations in the final achievement of renovations, succeed in various efficiency and appearance of the final improvement. Therefore, this paper proposed a comprehensive strategy to complete the renovation works towards energy saving and even achieve zero energy ready house in the future prospect. It provides an explore attempt on the existing contemporary building with century history, at the same time, it is expected to show another possibility to manage the sustainability retained in the local residential buildings. while acknowledging that incomplete assessments are used to justify demolition, or to layer fashionable “innovations”.

### The renovation project of Qingdao Liyuan buildings

Liyuan buildings are built based upon the European style courtyard style of 1900s. There are typical design features with court surrounded by two to three floors of buildings. This building styles were used as the main residential buildings in the urban planning accompanying the early time when the main urban area appeared its original appearance.

**Figure 1:**
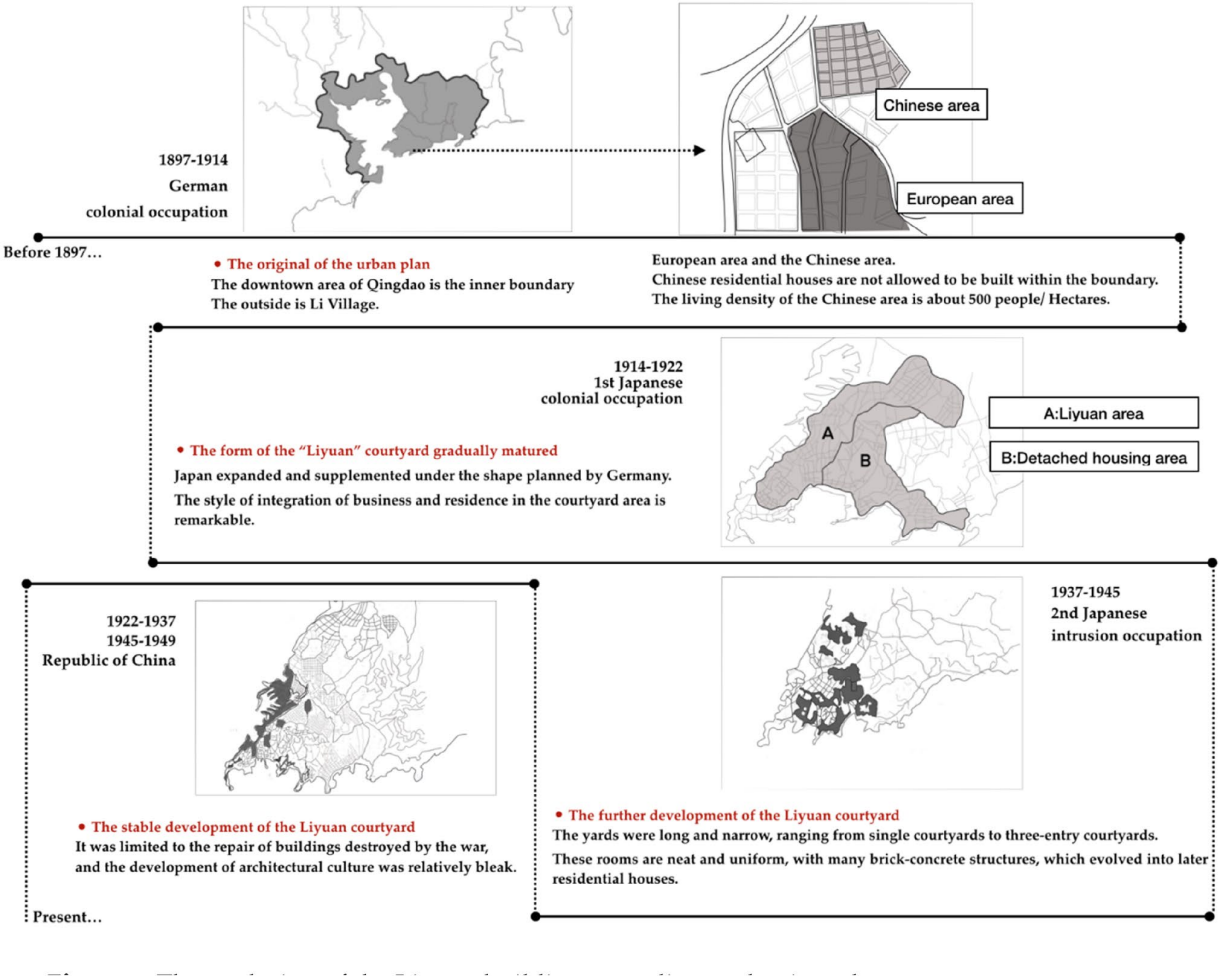
The evolution of the Liyuan building according to the time clue.

The Liyuan courtyards mentioned in this article refers to the buildings firstly built in the 1900s under the colonial rule by Germany intruders, which was retained its basic appearance till today (Fig. 1). Once upon a time, the Liyuan courtyard buildings spanned a century is still a typical representative symbol of the early urban construction of Qingdao. The "blocks" here are defined with boundary of urban traffic nets, including the neighboring buildings served functionally for the courtyards, contains internal plugging and corridors as well as social living spaces. Business and residence are mixed, shops and shops are intertwined. The "Li" concept in “Liyuan” was originally referred to the commercial function, which means the shops outwards to the streets for business negotiation by merchants. They can check goods samples when walked into the "Li” or collect products inside the "Li". On the other hand, the "Yuan" concept referred more specific in the living function, with larger scale than "Li". These two concepts were combined by government architectural department as the current definition of “Liyuan” courtyard only in 1999^21–23^. The Liyuan courtyard building is an important representative architectural style, it also represents a living form of middle-lower-level recognition in Qingdao at earlier period.

**Figure 2:**
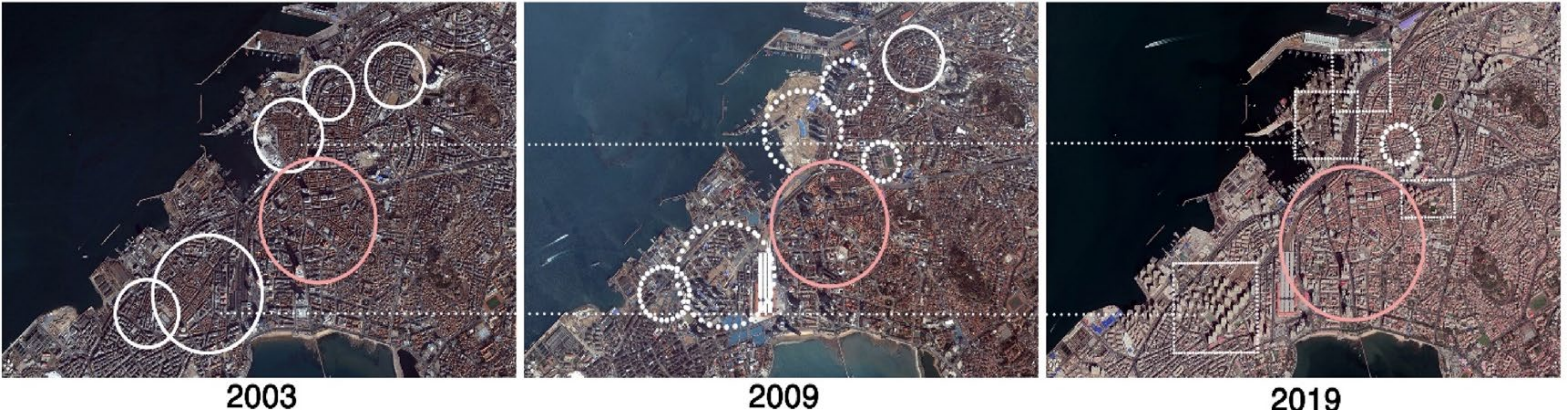
The shrinking of the distribution of Liyuan courtyard buildings in Qingdao.

Incredible development is pushing forward the great changes all over the world and many changes in human behavior results in ultra-large cities with million population in highly concentrated urban scale. To harness the energy potential of existing buildings is a mile stone to meet the targets for a low carbon economy in 2050^10^. Therefore, in China, the urban development is seeking the solution to achieve better energy performance in the limited land area with suitable actions on the existing buildings. Meanwhile, some traditional old town areas are shrinking and might be eventually disappeared^24,25^. Similar to that, the distribution of Liyuan courtyard building is turning to shrinkage trends in area size (Fig. 2).

As the purpose of upgrading the appearance of the city and improving the life quality for the domestic people, the municipal government of Qingdao started a series projects on exploration of the revitalization and urban renewal for the old town area from 2015. About 28 square kilometers of old town area are involved in this project, including a large number of Liyuan courtyard buildings protection and retrofit. With the general policy orientation of "Repair the old town and keep its originality, rely on industry-driven and remain technological empowerment", comprehensive assessments and renovation actions have been proposed in order to reform urban infrastructure construction, social livelihood and ecological improvement.

**Figure 3:**
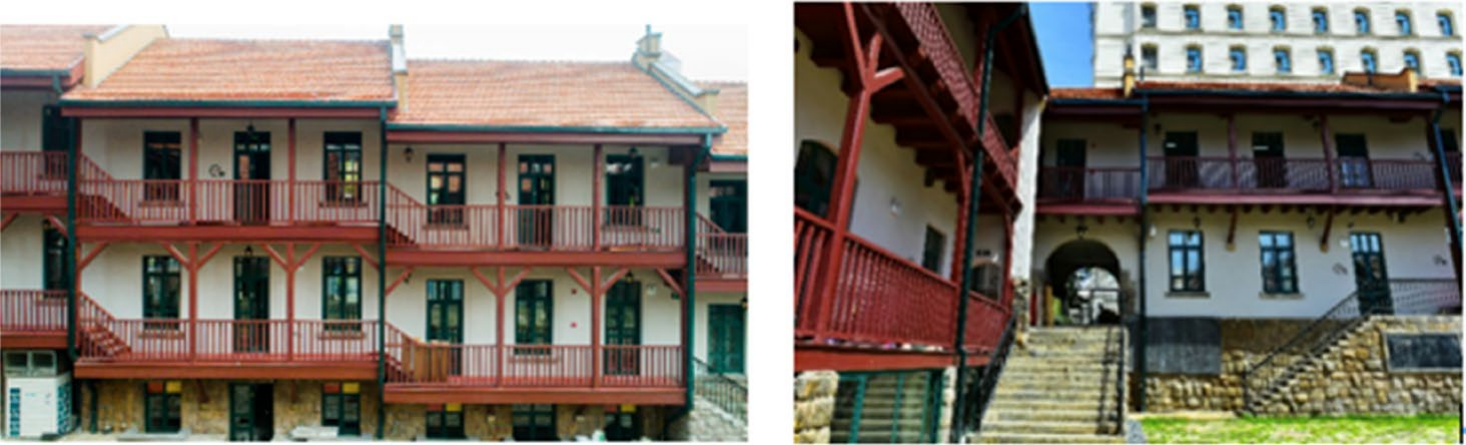
The authentic appearance of a typical Liyuan building example.

The general layout of the Liyuan courtyard consists of two sections, the outer building looks like the rows of noisy street shops (Fig. 3). While the inner part is filled with the private corners with daily lives. A courtyard separates crowed public from quiet corners. When the construction of an open, modern, dynamic and fashionable international metropolis becomes Qingdao's mission, this old building style become the new starting point for the revival of the history of the city. Although neighborhoods are composed of houses, communities are more than just sticks and bricks. They include and are formed by people and social relationships, both within and across houses. This was as true in old times as it is today.

It is far from satisfaction by implementation energy saving technologies and renewable energy sources alternatives only in the new built buildings. There is only small percentage of new built buildings in most of the countries all over the world, the large number of existing buildings with high energy consumption in use contains great opportunities to reduce global energy demand and pollution potential in the future. It agrees with the facts in EU project Annex 56^26^ that “the greenest buildings” is the one that is already built for long time^27^.

### The research objectives

This research is aiming to draw sustainable concept on the energy issues within existing contemporary buildings. Beginning with brief assessments in the case study, the physical and energy performance embedded is illustrated with parametric models and analyzed. With building performance simulations in software Ecotect, it calculated the energy performance in the total energy consumption and discussed motivations of different strategies through renovations and optimization options. It is expected to discover the sustainability embedded within the certain old buildings with feasible upgrades and to emphasize the sustainability and vitality in the architectural preservation. There are several goals to be observed in this paper:

1. Simulate multiple optimization scenarios and calculate thermal performance though computational simulations;
2. Evaluate energy saving potentials by comparing parametric results from simulations in renovation scenarios and analyze the energy improvement with suitable renovation.
3. Discuss the retrofit strategies on different energy efficiency actions in old buildings upgrade and benefits relatively, conclude the sustainability embodied within the comtemporary buildings from different perspectives.

## Materials and methods

There are two parts in this research. The first part focuses on finding out the parametric characteristics and dimensional data from observations and field investigation in the architectural features. With other collective data, such as meteorologic, building materials etc., the parametric models was built in Rhino SketchUp for visualization analysis. It is a direct reflection from static state. The second part focuses on the perspective of building energy from computational building performance simulation with all collective data input into Ecotect for further simulation. SketchUp is a 3D modeling computer program for drawing applications such as architectural, interior design and etc. Autodesk Ecotect Analysis is an environmental analysis tool that allows building performance simulation throughout the whole lifecycle covering every stage of conceptual design. The two programs combined analysis functions with an interactive display that presents analytical results directly within the context of the building model.

The enhancement in the building envelope and proposed common upgrade scenarios in the refurbishment were simulated and compared. The aim is to allow the actions shifting resources, maximizing development efforts on BIM solutions for building performance analysis and visualization. It supports the further discussion in sustainability through the energy performance simulation.

### Parametric model analysis from static dimensional investigation

Comprehensive field investigation is the most efficient measures to get insight of the building performance, while acknowledging that complete assessments are necessary to justify demolition before any actions of renovation on the heritage buildings. Through literature records study, the historical and physical development documents provide the image on the storyline embedded within the buildings. The detailed field investigation has been carried out from 2017, including external observation, internal assessment, random interview, dimensional measurement and illustration analysis etc.

There are several major parameters influenced the building energy performance analysis, including environmental, insulation and instruments installed. The geographical information decides the meteorological conditions of the area, which provided temperature, humidity and solar radiation etc. It determined the necessary heating and cooling demand and the possible options for refurbishment. The insulation is another important parameter influenced by the structure and materials. Great improvement in materials provided better efficiency and possibilities to reduce the energy consumption and achieve sustainable goals. It is also considered as the recent research focus. New type of instrument with lower energy consumption and green energy supply could supply new developing points in sustainable building performance.

The research of refurbishment would be mainly focus on the insulation improvement here. In order to illustrate the structure and show up the inner design within these Liyuan residential buildings, a digital drawing describing dimensional details was conducted after survey studies. The dimensional information has been visualized to provide a brief look into the facts of these existing buildings throughout hundred years history.

### Renovation scenarios and iterative sustainability analysis

It is necessary to fulfill the local architecture building standard before reasonable sustainability analysis of the renovation actions. In China, JGJ26-2018 is published as the green building renovation goal for the renovation standard in Qingdao located in the cold climate zone^28^. Therefore, one scenario of renovation is to fulfill the green building standard for the whole building scale, which contains the consideration of local climate condition and majority of local heating demand and living habits. The other scenario of renovation is to mix part of the exterior insulation layer with recycling concrete materials, in order to discover the energy saving potential embedded within the traditional buildings. Optional alternatives with renewable energy, such as solar panels, taken as for heat demand resources will be discussed as specific supplement choice to the energy supply.

**Figure 4:**
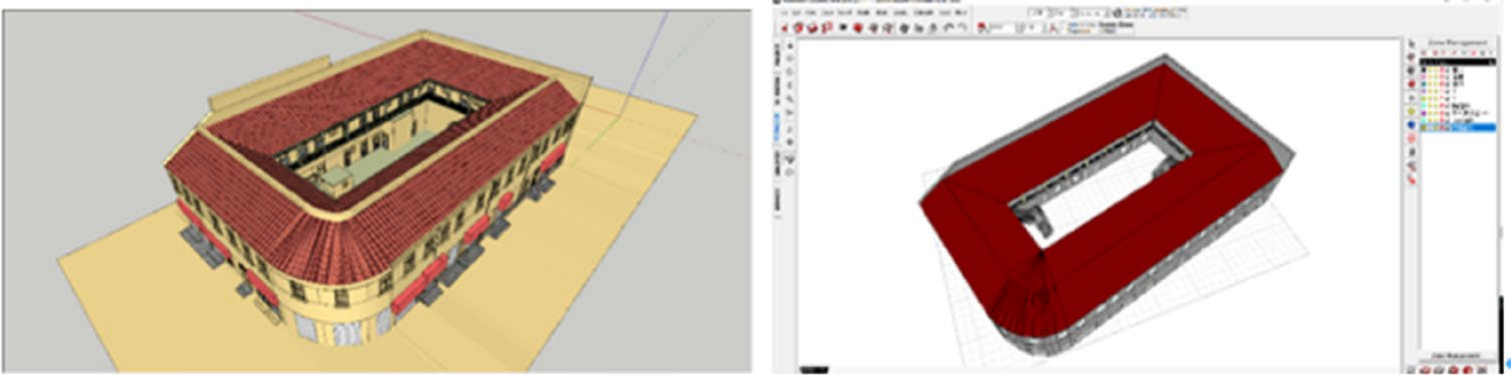
The building models (left) and performance simulation through Ecotect software (right).

Energy consumption within complicated procedure of renovation requires high quality of modeling effort from captured building data and keep updating, uncertainty of information, objects and relations among software data is a big challenge. Meanwhile, it is agreed that the building performance simulations (Fig. 4) can be great helpful to engage the interests from different stakeholders based on their individual background knowledges with intuitive and direct images. Visualization tools empower owners and users with a better understanding of their house’s physical behavior but have limited effect on their renovation decisions.

With hypothesis of certain potentials existing in the static and dynamic states, it expected the results would give more impressive imagination of the listed building and old contemporary building when restoration would be the energy improvement embodied this characteristic building structure. It is expected to attract potential stakeholders and users to realize the positive impact from the process of renovation for heritage buildings, meanwhile, to consider and encourage more active positive policy on the sustainability in the building retrofit and use. The results and findings from this paper are extension of the knowledge for all potential stakeholders, to consider the old contemporary buildings as architectural heritage from both static and dynamic states, to broader their awareness on the energy properties in accordance with modern standard.

There are certainly limitations existed in the simulation procedure. The results from building simulation could be only suitable to the certain situation in the case study with specific local information. The complicated building performance is simplified and focus on the energy parameters in the data collection and calculation procedures.

### Ethics approval and consent to participate

This manuscript does not involve any animals, humans, human data, human tissue or plants.

### Consent for publication

This manuscript does not contain any data from any individual person.

## Results

**Figure 5:**
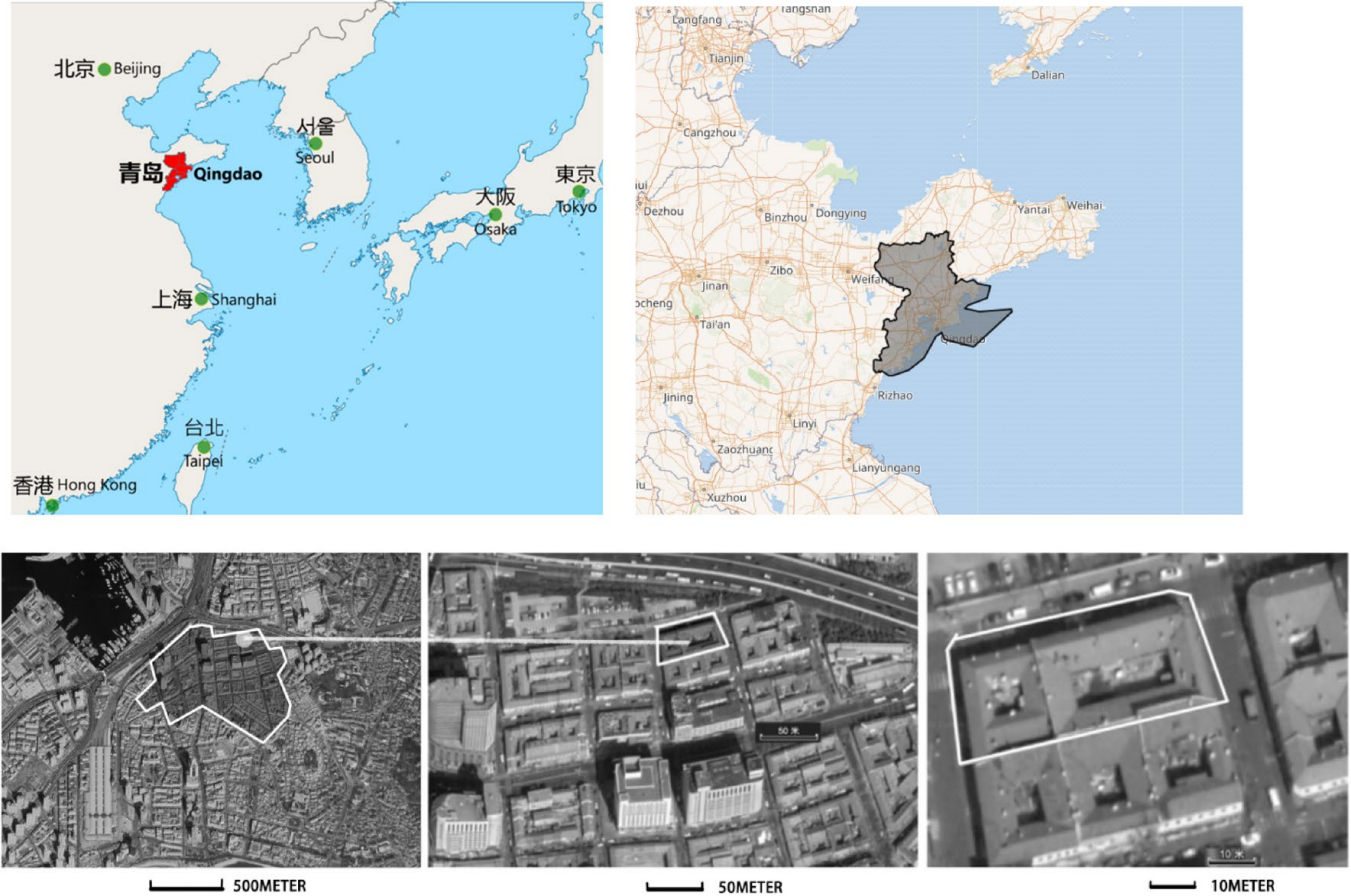
The location of the Liyuan courtyard buildings of the case study in Qingdao on the map.

In this paper, the dual-perspectives assessment method was carried out in a case study located in the No. 84 of Zhifu Road, which is called “ZF-84” for short. The building locates in the highlighted area at the northern boundary of the original distributed area (Fig. 5). The building is in the block surrounded by Licun road, Yizhou road, Jimo road and Zhifu road, underneath the newly built elevated road.

This ZF-84 building is a two-stories with traditional German style, installed red tiles on top and half-timbered brick structure, from the initial records in historical document and the dimension measurement collected in documents collection, the original land occurrence in the very beginning was 838.74 m^2^, while the current is 968.23 m^2^; The original building area was 1695.91 m^2^, while the current is 1825.40 m^2^ (Table 1).

**Table 1:** The dimensional information of the building.

No. of floors	2	
Total floor area	1310 m^2^	
Window area	66 m^2^	
Atrium area	273 m^2^	
Location	Qingdao, China	
36° 5′ N, 120° 20′ E		
Orientation	Main façade oriented 20° to northwest	

The building was used to be an iron blacksmith store in the early 1930s, which was further modified as an iron plant, together with other surrounding workshops, which was recognized as the predecessor of “Qing gang” Iron factory. It was the most glorious time for this building. After the war time, it was changed as an Asian-American firm store selling skin care products, and also a workshop producing thread rolls. There was some maintenance in partial elements in the 1980s. The original wooden handrails and stairs were changed to stone ones at that time. The pillars used to be made of iron was also replaced by wood materials. There happened other restoration actions in the roof tiles, but it only focused on spray painting on the façade, appearing as a new one from outside. To get rid of the leakage of rain water, during that time, the residents built some personal facilities on their own tiles and added handrails of stairs with cement.

### Demonstration of architectural features

The general judgement of the current condition described the building in a bad condition mostly due to the devastated along the long time. There is limited living area in each room space and narrow access available in accordance with the modern living condition, even less public space than imagine due to plenty private additional facilities in the atrium yard. There are obvious defects from the initial observation, which are opponent to the modern comfort demand in the living environments.

With the help of architecture software, the results from investigation on current spatial situation of the buildings are analyzed following different functions for detailed analysis, which focused on the building plans, public areas, function areas and added areas (as shown in Table 2). Different colors are labeled in the planning drawings, which are used to explain the relationship between different space area.

**Table 2:** The layout analysis of the building.

Detailed information	1st floor	2nd floor	Memo
Plan			
Public area			
Function area			
Added part			

**Figure 6:**
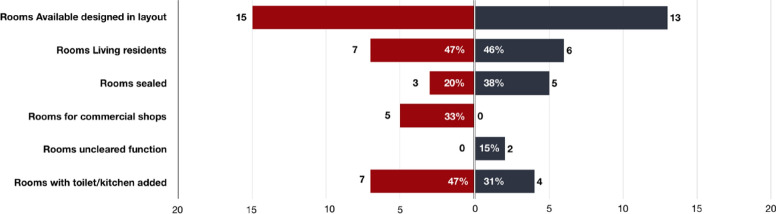
Spatial analysis of rooms in the case study building ZF 84 (Red for 1st floor; Black for 2nd floor).

The statistical database was summarized based on architecture features from the plan layout analysis in Table 2. There are totally 28 families still living in the building based on the statistical data collected in 2019. The architectural distribution here are mostly changed by private additional facilities building to meet their basic requirements (Fig. 6).

### Simulation of energy performance

For the energy perspective, there are two comparative scenarios analyzed in the building performance simulation, see Table 3. The original performance of the building labeled as “Model_1” is referred to the original built reference for the listed building, which induced the single glazed timber framed windows, wooden doors, old clay tile roofs, and brick timber framed walls. “Model_2” is one scenario with modification in accordance with conservation requirement standard implemented double glazed windows, exterior walls of insulation and renewal of roofs and doors to improve the thermal properties and physical behavior of the building to fulfill the conservation requirement. “Model_3” is another model implemented with suggested modification by replacement of recycling construction materials^29^ in envelope wall and further optimization in other building components. Both renovation scenarios are going to improve the whole building energy saving goals to achieve the green building standard and even higher efficiency.

**Table 3:** Comparative review of the building envelope in terms of thermal protection and the annual energy demand, checked through simulation in the ZF case study**.**

Model		Model_1	Model_2	Model_3
Description		The listed building in current situation	The modified building in accordance with conservation requirement	Further modification with suggested changes
Windows		Single glazed timber framed	Double glazed windows	Double glazed windows
U (W/m^2^K)		5.100	2.000	
Door		Single layer wooden	Single layer wooden	Enhanced wooden doors
U (W/m^2^K)		2.310		2.260
Roof		Old clay tile	Renewed tiles	Renewed tiles
U (W/m^2^K)		3.100	0.260	
Walls		Brick timber framed	Insulation walls	Insulation walls with recycling materials
U (W/m^2^K)		1.200	0.300	0.230
Energy demands	Heating	112,547.288	65,919.164	64,760.580
	Cooling	8504.943	13,056.007	13,749.030
	Total	121,052.231	78,948.171	785,093.610

With suggested analysis on the building energy perspective, comparative review on the three analyzed models of the ZF building in the case study explain how it is working and possible changes with the common suggested refurbishment measures in the terms of energy (Table 3) through the building performance simulation. The calculation of the energy consumption didn’t include the associated facilities because the lighting and appliances were installed separately according to the individual intentions.

The thermal transmittance (U-value) of the materials in different construction elements for the three models were calculated according to the National green building standard in the cold zones of China (2019) and the materials properties were obtained from the local design guidance for construction. The U-value of the possible energy refurbishment measures are also shown in Table 3.

Considering the rate of use of the equipment, lights and occupancy are the same in the three models, the most indicative aspect is the heating and cooling energy demand. From the results of the energy performance simulating presented in Table 3, it is obviously the Model_3 outperforms a little bit better than the ordinary in Model_2 and it is a significant improvement of the baseline in Model_1.

**Figure 7:**
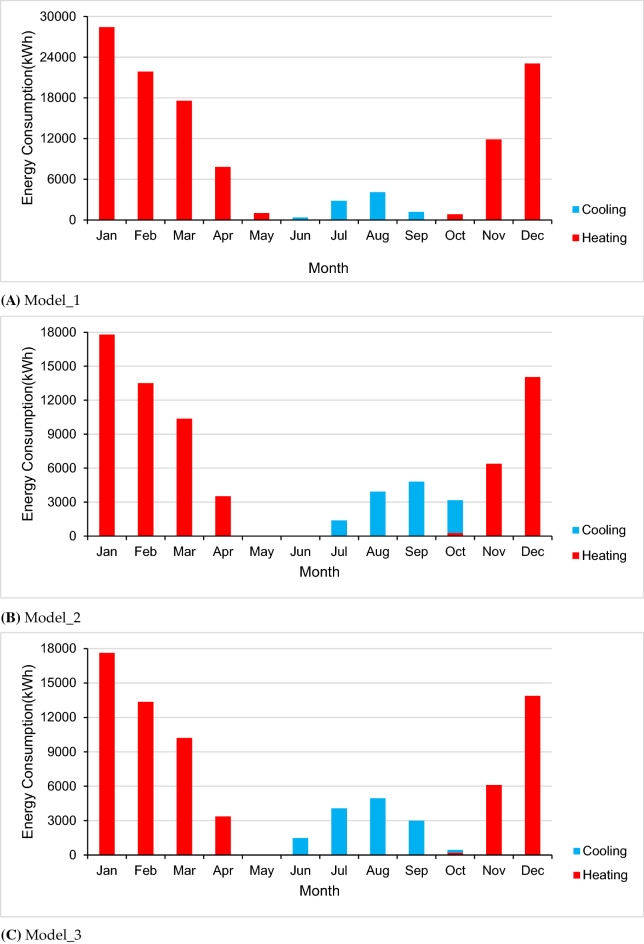
Monthly energy consumption of the models. (**A**) Model_1; (**B**) Model_2; (**C**) Model_3.

The energy performance of the three models throughout the year is shown in Fig. 7. The peak heating demand occurred in period from December to February, the peak cooling demand happened in July to August period. Compared to the peak value of heating demand in the Model_1, it decreased to 24,700 kWh in the Model_2 which was 11.8% reduction, while the value in the Model_3 was decreased to 24,550 kWh which was approximately 12.3% less than the number in Model_1, and even 1% less compared to Model_2 in the same weather condition. There is not a long season required cooling demand, the peak values in cooling demand of all the three models happened around August and have big various due to different modification. But generally, the cooling demand is a slightly increased after insulation modification, but still small percentage compared to the heating demand over the year.

**Figure 8:**
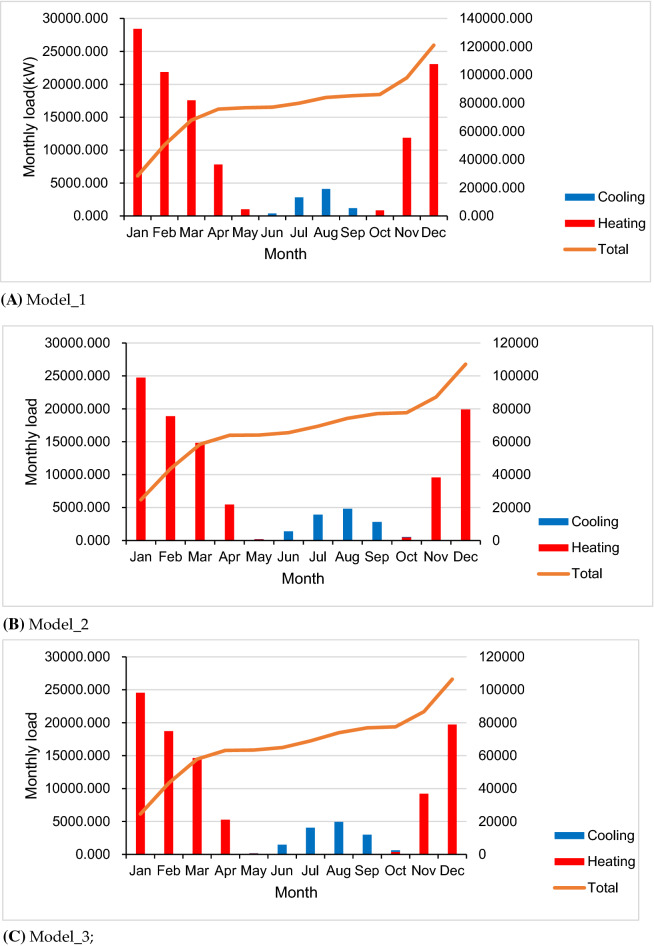
Monthly energy load profile. (**A**) Model_1; (**B**) Model_2; (**C**) Model_3.

The monthly heating loads variation are displayed in Fig. 8. It is evident the heating loads in Model_2 and Model_3 was significantly reduced than the Model_1 in the winter period. While the cooling demand was increased slightly due to the better insulations retained better thermal properties.

There might also certain limitation in the results, because the building is old and most part are under maintenance. There almost too few people to check the validation of the building. However, the simulation and calculation results are expected to aid the stakeholders understand the building performance through different restoration strategies.

## Findings and discussion

Every city has its unique historical culture standing for its soul, it is specifically shown in the physical space among the historical blocks and daily life forms of the citizens live in it. As a typical buildings style with 100 years history combining Western and Chinese culture, the Qingdao Liyuan building request a better comprehensive assessment in accordance with modern living standard. According to the dual-perspectives analysis, the deep assessment on the Liyuan buildings is examined and explained from the two perspectives.

### Analysis in architectural features

**Figure 9:**
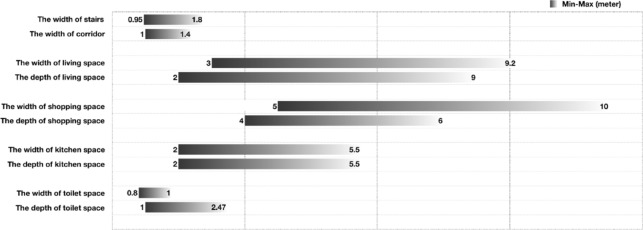
Statistical analysis in the spatial information in case study buildings.

A general spatial analysis of the rooms was conducted focused on various building facilities inside the buildings according to the field measurements data. As shown in Fig. 9, the minimal to maximum dimensional information was illustrated, it summarized the general features in this Liyuan courtyard building. (1) There is limited access space as the stairs width are from 0.95 to 1.8 m and corridor width are 1–1.4 m; (2) The living space is relatively spacious compared to the commercial space which is normally narrow while the space is generally small in size; (3) the kitchen space is average in size but very little sanitary space arrangement as the dimension in toilets are quite small even smaller than the access space. In general, the rooms in the Liyuan courtyard buildings are quite old-fashioned with small size for living and little space for access and sanitary and kitchen.

There are obvious defects existed in the Liyuan building design which opposed to the development trend of modern comfort in the living environments.

1. Bad sanitary condition. Only one or two public toilets arranged in the original design in this courtyard for all the residents which reflect the outdated sanitary prohibitions and class discrimination to Chinese workers for low residential standard in the colonial time.
2. Insufficient facilities served. No associated facilities supporting daily lives to meet the privacy and space requirements of the modern living style.
3. Small spatial arrangement in the rooms. The space allowance is limited to the minimum living needs, unable to meet more public and flexible areas.

### Analysis in energy performance features

The energy performance is always complicated involving considerations on many factors. The analysis through building performance simulation with concerns on the energy performance tried to give deeper impression on the building from dynamic state.

**Figure 10:**
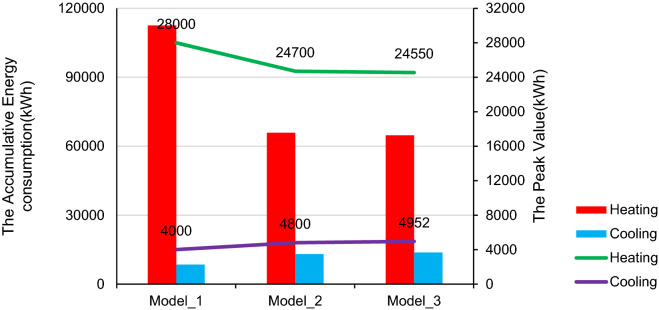
The variations in the heating and cooling consumption from the accumulative amount and peak value.

With a comparative analysis of the peak values variation in the heating and cooling demand of the simulation models (Fig. 10), the upgrading of thermal insulation and materials improve the building performance greatly. The heating load of the building in the cold season is greatly reduced, saving nearly half of the heat energy. While the cooling load slightly increased during summer period. It shows that changing the thermal performance of building exterior envelope materials can effectively improve indoor comfort and reduce energy consumption in severe cold seasons, but it will also bring negative effects slightly in hot seasons.

Meanwhile, the data shows that although the energy consumption in the hot season has increased, from a year-round perspective, the heat consumption in Qingdao is several orders of magnitude higher than the cold consumption. The amount of energy saved by increasing the thermal performance of building exterior walls is still considerable. Therefore, it is still a very efficient measure to transform the thermal performance of listed building.

**Figure 11:**
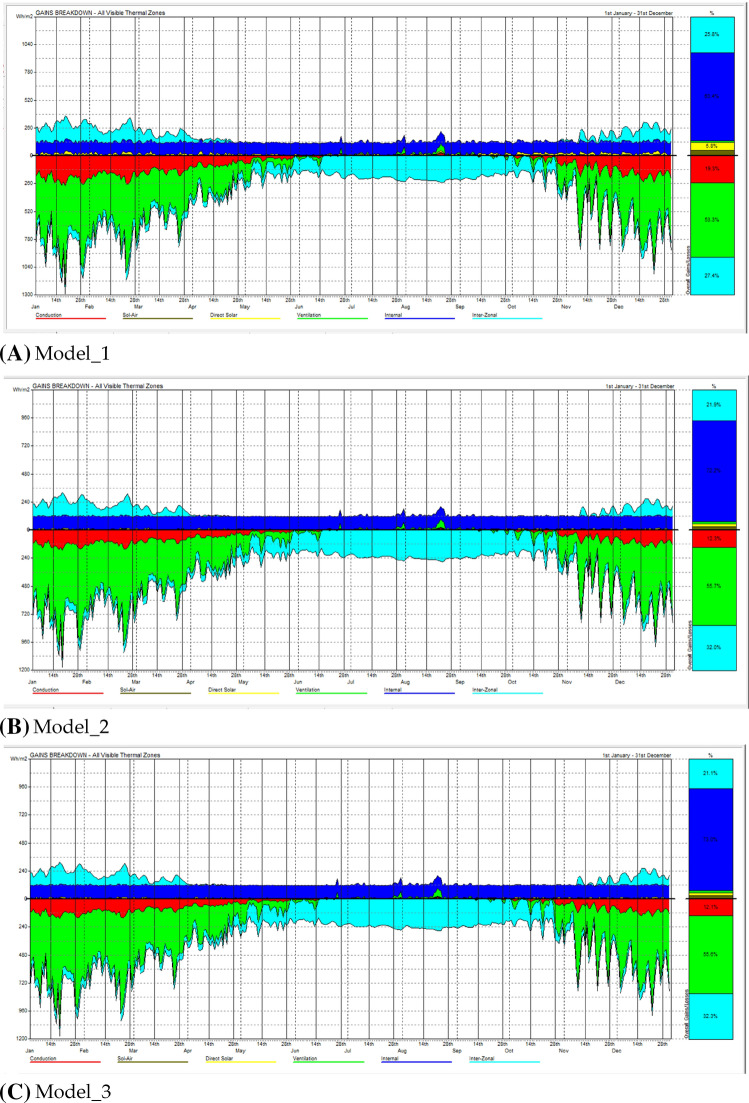
The passive grains breakdown analysis. (**A**) Model_1; (**B**) Model_2; (**C**) Model_3.

The passive grains breakdown distribution displayed in the Fig. 11 illustrate more consideration on the energy perspective. There are several considerations from the pictures.

1. The annual heat loss is much higher than the income in the listed building, thus, heat preservation in winter will be far more energy-saving than heat dissipation in summer.
2. The three models all start from improving the thermal functionality (K value) of the building’s external walls, which succeed in reducing the heat loss caused by heat transfer from 19.3% of the total loss to 12.3% and 12.1%. It is very effective and gradually reduces the energy consumption of the building. However, heat loss from ventilation and heat dissipation is still a drop in the bucket. Therefore, in the conservation of Liyuan building, not only the thermal performance of the material itself, but also the design correction on airtightness of the interior and exterior spaces of the building should be handled well.

Related to Qingdao Liyuan building style, the existence of the courtyard increases the building surface area, and the short depth in the rooms also makes it relatively easier access the wind to take away heat from the inside. The general wooden structure of the listed building and wall have high coefficient of expansion, porosity, and poor airtightness, which also increase the ventilation heat loss to a certain extent.

Obviously, the selection of building wall insulation materials has a great impact on building energy conservation. At present, the building wall insulation performance of Liyuan buildings in China is poor, resulting in relatively high heating and air conditioning load of buildings in China, that is, under the same indoor environment, the building energy consumption demand per unit area is relatively high. By increasing the thickness of the external wall and reducing the heat transfer coefficient of the external wall, increasing the thickness of the external wall could increase the weight of the external wall and consume a lot of building materials. Therefore, in energy-saving buildings, the heat transfer coefficient of the external wall could be generally reduced by reducing the heat transfer coefficient of the material and improving the structure.

### Sustainability embodied within the contemporary buildings

The existing Liyuan buildings are strongly demonstrate resilience and versatility, their capability to persist throughout long time should be appreciated as a symbol of sustainability. The demand to preserve such heritage buildings and prevent further decay, while preserving embodied unique historical characteristics provide extended perspectives in the project of retrofitting. By protecting the authentic brick façade and maintaining the continuity of the original purpose of the building, it initialized more consideration on other perspectives besides the improvement of the energy performance.

The proposed dual-perspectives method on assessment of retrofitting upgrade was not only focused on the authentic visual appearance, but also estimate the energy performance through reasonable simulations based on the information collected. This can help to verify the extent of how the retrofitting meet the living standards on energy efficiency and comfort conditions comprehensively.

The simulations in this case, analyzed and explained adequate renovation measures with statistical data. By empowering the envelop properties enhancement, it is obvious to gain better energy efficiency and thermal performance in heating demand during winter, while get certain loss in cooling demand during summer. This could be further improved according to the frequency of use of the room in different seasons, so as to adjust the heat energy consumption as much as possible while keeping the cold energy consumption in a stable level.

The proposed upgrade measures reduced the thermal loss from 19.3 to 12.3%, which increased energy efficiency greatly. It is evident the building energy efficiency can be improved with adequate measures, with certain careful consideration of necessary premises for the existing contemporary buildings as following conditions:

- Use the environment friendly materials to get rid of the possible embodied emission.
- Execute comprehensive analysis of the current conditions of the building.
- Determine the restoration goals in different perspectives and the extent.
- Decide suitable construction measures to ensure the energy retrofitting goals and maintain the original function following the guidance in the conservation requirement.
- Estimate the energy efficiency with reasonable simulation to help making decisions on the choice between different methods, to obtain the general judgement on the building conservation in full-scale.

It is suggested for all the architects to better understand the old contemporary buildings from multi-perspectives, especially with more consideration in the combination of architecture design with energy performance. With the aims to overcome the energy crisis and reduce heat losses, it is necessary to apply adequate thermal insulation in the building envelope elements, which is the most effective measures because the devastated building cannot ensure thermal properties in good condition. While respecting the conservation requirements and maintaining the authentic appearance of the listed building, various models of restoration were suggested and analyzed through building performance simulation (BPS) method. The results and findings of the paper verified the success in the energy efficiency improvement within the example in Qingdao Liyuan buildings, and it contributed with practical certification by applying the principles and retrofitting measures in the restoration project.

This individual approach is necessary in every old contemporary building to ensure the realization of restoration in all the perspectives could be implemented with satisfying results. It suggested the knowledge gained from this case study would serve as support for possibility discussion of specific strategy explore based on the guideline upgrade of the whole neighborhood scale in the retrofit of old buildings in a more comprehensive extent.

## Conclusions

This paper investigated the sustainability embodied within a typical contemporary residential building date back to 1900s in China, which still exists nowadays as symbol of traditional culture and classical architectural style. The research is going to consider how the initial residential demands could be satisfied with reasonable renovation actions under energy consumption optimization direction. Field investigation and deep assessment on aesthetic succession were proposed as triggers for new target toward better energy efficiency in building performance. Two scenarios executed in the simulation of case study towards lower energy consumption purpose. This environment friendly practice could fill up the gap between excessive natural resource and limited requirement for architecture buildings. To sort out the internal meaning and information of the traditional architectures in the backward time period, it can be great help to understand how unique they are to maintain detailed records of its characteristics and recover its brightness with the modern technologies. The comprehensive result from the survey investigation in the case study proposed a feasible and ecological design database for the retrofit and improvement of Liyuan courtyards.

Associated with the specific case studies, intuitive descriptions make representations of the reality attractive and understandable by others beyond specific areas of knowledge. Limitations like the difficulty to read plans and cuts, to anticipate cost scenarios and to include holistic complexity find in these models the multidisciplinary convergence needed for applied interdisciplinary studies. Most Liyuan courtyard buildings in Qingdao still have it basic functions for use, and they can somehow retain distinctive architectural forms and pleasant street environment through reasonable renovation and retrofit. As an excellent supplement to tourism resource, this can be valuable attempt to recover the original characteristics and charms by this practice of retrofit and refurbishment operation on the existing Liyuan courtyards. The purpose of reviving the historical architecture can be realized by the careful retrofit constructions. This attraction from history presented as a new form of architectural retrofit will be another achievement of sustainability in the economic manner.

Energy perspectives assessment provided an opportunity to get deeper insight into more detailed information embodied within the contemporary buildings. There are possible measures to improve the living comfort by changing the arrangement of the rooms and purpose of using, which may change the using time and occupancy in the building and furthermore change the human behavior inside the building. By applying insulation or changing the glazing windows and other various measures, the building energy performance could be improved and achieve the modern green building standard. In conclusion, this dual-perspective method provided a chance to explain the old buildings with better acknowledgement within the building and require the architects to apply more comprehensive consideration in the design phase, and get better retrofitting measure in the project of individual contemporary building.

## Data Availability

The datasets during and analyzed during the current study are available from the corresponding author on reasonable request.

## References

[1] Rao P (1999). Sustainable Development.

[2] United Nations. About the sustainable development goals. *United Nations Website*. https://www.un.org/sustainabledevelopment/sustainable-development-goals/. Accessed 19 August 2021.

[3] Meggers F (2012). Reduce CO_2_ from buildings with technology to zero emissions. Sustain. Cities Soc..

[4] Mazria, E. Resusitating a dying world, the 2010 imperative, a global emergency teach-in. *Architecture 2030* (2007).

[5] Roodman D, Lenssen N, Peterson J (1995). A Building Revolution: How Ecology and Health Concerns are Transforming Construction.

[6] U.S. Green Building Council, LEED rating system. *U.S. Green Building Council Website*. https://www.usgbc.org/leed/. Accessed 19 August 2021.

[7] D’agostino D, Zangheri P, Castellazzi L (2017). Towards nearly zero energy buildings in Europe: A focus on retrofit in non-residential buildings. Energies.

[8] Sustainable Japan. ZEH *Sustainable Japan News Webpage*. https://sustainablejapan.jp/2016/10/10/zeh/23955 (2016).

[9] Huang B, Mauerhofer V, Geng Y (2016). Analysis of existing building energy saving policies in Japan and China. J. Clean. Prod..

[10] Commissie E (2011). A Roadmap for Moving to a Competitive Low Carbon Economy in 2050.

[11] Staffansson Pauli K, Liu J, Bengtsson B (2020). Sustainable strategy in housing renovation: Moving from a technology-and-engineering-focused model to a user-oriented model. Sustainability.

[12] Mwasha A, Williams RG, Iwaro J (2011). Modeling the performance of residential building envelope: The role of sustainable energy performance indicators. Energy Build..

[13] Risholt B, Time B, Hestnes AG (2013). Sustainability assessment of nearly zero energy renovation of dwellings based on energy, economy and home quality indicators. Energy Build..

[14] Vieites E, Vassileva I, Arias JE (2015). European initiatives towards improving the energy efficiency in existing and historic buildings. Energy Proc..

[15] Versaci A (2016). The evolution of urban heritage concept in France, between conservation and rehabilitation programs. Proc. Soc. Behav. Sci..

[16] Asadi E (2012). A multi-objective optimization model for building retrofit strategies using TRNSYS simulations, GenOpt and MATLAB. Build. Environ..

[17] Chang N-B (2010). Systems Analysis for Sustainable Engineering: Theory and Applications.

[18] Juan Y-K, Gao P, Wang J (2010). A hybrid decision support system for sustainable office building renovation and energy performance improvement. Energy Build..

[19] Allen DT, Shonnard D (2012). Sustainable Engineering: Concepts, Design, and Case Studies.

[20] Nelson, B. *et al*. From the 16th to the 21st century: Upgrading Traditional Knowledge to approach Net Zero goals in existing neighborhood upgrades (ZERH-NU). ePrints at https://discovery.ucl.ac.uk/id/eprint/10075861/ (2016).

[21] Li D, Xu Y (2003). Three forward leaps in Qingdao’s urban development history: More discussion on the relationship between urban planning and urban development. Urban Plan. Forum.

[22] Li D, Xu Y (2006). Historic base of contemporary Chinese urban planning ideology in view of the development of Tsingtao's modern urban planning. Urban Plan. Forum.

[23] Zhang M, Rasiah R (2013). Qingdao. Cities.

[24] Long Y, Gao S (2019). Shrinking Cities in China: The Other Facet of Urbanization.

[25] Long Y, Wu K (2016). Shrinking cities in a rapidly urbanizing China. Environ. Plan. A.

[26] Nelson, B. “Montarroio Shinning Example” for “IEA EBC Annex56 Shinning Examples publication”: Residential Building Upgrade in Montarroio. ePrints at 10.13140/RG.2.1.1029.9600 (2015).

[27] Frey P (2011). The Greenest Building: Quantifying the Environmental Value of Building Reuse.

[28] He Q (2020). A data-driven approach for sustainable building retrofit—A case study of different climate zones in China. Sustainability.

[29] Yongquan H, Zheng W, Leji L (2018). Research on basic performance of recycled aggregate of construction waste. Recycl. Resour. Circ. Econ..

